# Successful management of a rare extensive hepatopulmonary hydatidosis with over 35 cysts: A case report and literature review

**DOI:** 10.1016/j.ijscr.2025.111807

**Published:** 2025-08-14

**Authors:** Salahaldeen Deeb, Dana Sayyedahmad, Zaid Sharif, Sireen Manasrah, Hussein Abusharkh, Yousef Abuasbeh

**Affiliations:** a Faculty of Medicine, Al-Quds University, Jerusalem, Palestine; b Faculty of Medicine, Alexandria University, Alexandria, Egypt; c Radiology department, Al Ahli Hospital, Hebron, Palestine; d Thoracic Surgery Unit, Al Ahli Hospital, Hebron, Palestine

**Keywords:** Pediatric hydatidosis, Hydatid cyst, Minimally invasive, Case report

## Abstract

**Introduction and importance:**

Extensive hepatopulmonary hydatidosis is a rare and challenging manifestation of *Echinococcus granulosus* infection in pediatric patients. It can lead to significant morbidity if not promptly recognized and appropriately managed. Early diagnosis and a staged multidisciplinary approach are essential to achieve favorable outcomes.

**Case presentation:**

An 8-year-old boy presented with weight loss, cough, and hepatomegaly. Imaging revealed over 35 hydatid cysts in the liver and both lungs. He underwent staged bilateral lung cyst excision using hybrid minimally invasive surgery, followed by PAIR procedures for liver cysts and albendazole therapy. Postoperative recovery was uneventful, with full resolution of lung cysts and significant reduction in hepatic cyst burden.

**Clinical discussion:**

Extensive multi-organ hydatidosis in children is rare. Management depends on cyst location, number, and complications. Minimally invasive surgery combined with medical therapy is effective.

**Conclusion:**

This case highlights the importance of timely diagnosis and staged multidisciplinary management in pediatric multiorgan hydatidosis.

## Highlights

• Exceptionally High Cyst Burden in a Child: This case involved over 35 hydatid cysts across the liver and lungs, making it one of the most extensive pediatric hepatopulmonary hydatidosis cases reported.
• Rare Multiorgan Involvement in Pediatric Hydatidosis: Simultaneous hepatic and bilateral pulmonary cysts are exceedingly rare in children, posing significant diagnostic and therapeutic challenges.
• Minimally Invasive Hybrid Surgical Approach: The use of video-assisted thoracoscopic surgery (VATS) combined with mini-thoracotomy enabled effective lung cyst removal with minimal complications.
• Effective Use of PAIR and Albendazole: Sequential PAIR procedures and postoperative albendazole therapy significantly reduced hepatic cyst burden, highlighting a safe and effective non-surgical strategy.
• Successful Multidisciplinary and Staged Management: A well-coordinated, staged treatment plan led to complete pulmonary recovery and substantial hepatic improvement, underscoring the value of a multidisciplinary approach.

## 1 Introduction

Hydatid disease, caused by *Echinococcus granulosus*, is a global health concern, particularly in endemic regions like the Mediterranean, the Middle East, and parts of South America [1]. The liver is the most commonly affected organ, followed by the lungs. Concurrent liver and lung involvement is relatively rare, occurring in 4.6 % to 12.4 % of cases [2].

The disease thrives in areas with extensive sheep farming, facilitating the life cycle of the *Echinococcus* parasite [3]. Canines serve as definitive hosts, livestock are intermediate hosts, and humans become accidental hosts by ingesting the parasite's eggs [4]. These eggs hatch in the intestine, and the larvae penetrate the intestinal wall, entering the bloodstream to migrate to the liver and lungs, where they form cysts.

Hydatid cysts grow slowly, so the disease can remain asymptomatic for years. When symptoms occur, they are non-specific and depend on cyst location. Liver cysts may cause abdominal pain, nausea, or hepatomegaly, while lung cysts can lead to cough, chest discomfort, or hemoptysis [4]. Diagnosis is confirmed through imaging techniques like ultrasound, CT, or MRI, which detect characteristic cystic lesions. Serological tests can assist but are generally less specific.

Treatment typically involves surgery and anti-parasitic therapy. Surgery aims for complete cyst removal without spillage to prevent anaphylaxis or disease spread [1]. The approach depends on cyst size, location, and complications. Hepatic cysts may be managed with cystectomy, pericystectomy, or hepatic resection, while pulmonary cysts might require lobectomy or cyst enucleation. Anti-parasitic drugs like albendazole are used before and after surgery to reduce recurrence risks and treat residual disease. The prognosis is generally favorable with appropriate management [5]. However, recurrence is a considerable risk, especially if complete cyst removal is not achieved. Long-term follow-up is crucial to detect recurrences early and address potential late complications.

In this case report, we present an 8-year-old child with extensive hydatid disease involving 35 cysts distributed between the liver and both lungs. Such widespread involvement is exceptionally rare and emphasizes the challenges in managing multiple organ hydatidosis. This case highlights the importance of a multidisciplinary approach in effectively treating concurrent pulmonary and hepatic hydatid cysts. Through careful surgical planning and adjunctive therapies, the patient achieved excellent outcomes, demonstrating that even complex cases can be successfully managed with optimal intervention strategies, This case report has been reported in line with the SCARE 2025 criteria [19].

## 2 Case presentation

An eight-year-old Palestinian boy presented to the emergency department with a two-month history of progressive weight loss and a nonproductive cough. He had no significant past medical or surgical history and no known exposure to infected individuals or recent travel. His family lived in a rural area with multiple pets, including sheep, dogs, and chickens, raising the possibility of zoonotic transmission.

On examination, The patient weighed 21 kg and measured 118 cm in height, corresponding to below-average percentiles for age, consistent with chronic illness and nutritional compromise. These measurements were critical in planning anesthesia and port placement for the minimally invasive procedure. The child appeared well but had a mildly hyperemic throat and asymmetrical vesicular breath sounds. Vital signs were within normal limits except for a respiratory rate of 34 breaths per minute. Abdominal examination revealed tenderness with the liver edge palpable 17 cm below the costal margin and a liver span of approximately 13 cm extending into the epigastric area. These findings prompted further investigation due to the suspicion of hepatic pathology as shown in Table 1.

**Table 1:** Laboratory Results on admission.

Test	Result	Normal range	Comments
White Blood Cell Count (WBC)	19.45 × 10^3/μL	5.0–10.0 × 10^3/μL	Elevated
Hemoglobin (Hb)	10.79 g/dL	11.5–15.9 g/dL	Low
Platelets (PLTS)	542.4 × 10^3/μL	150–450 × 10^3/μL	Slightly elevated
Neutrophils	10.99 × 10^3/μL	2.5–7.5 × 10^3/μL	Elevated
Eosinophils	0.93 × 10^3/μL	0.04–0.44 × 10^3/μL	Elevated
C-reactive protein (CRP)	57.5 mg/L	Up to 6 mg/L	Highly elevated
Echinococcus Antibodies	Positive	Negative (< 1:8)	Abnormal

**Note:** All other laboratory parameters, including liver enzymes, renal function, and electrolytes, were within normal limits.

**Fig. 1:**
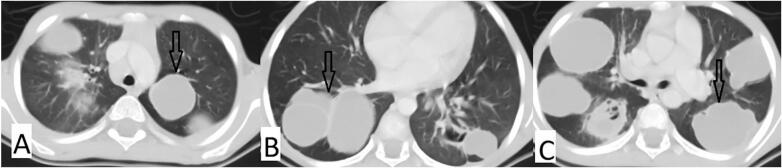
Presents axial CT images (A-C) at different levels of the lungs, each showing multiple bilateral well-defined hypodense non-enhancing cystic lesions with thin enhancing walls scattered throughout both lungs marked by black arrows, classified as CE3a. The largest lesion in the right lung measured about 5.5 × 4 cm.

**Fig. 2:**
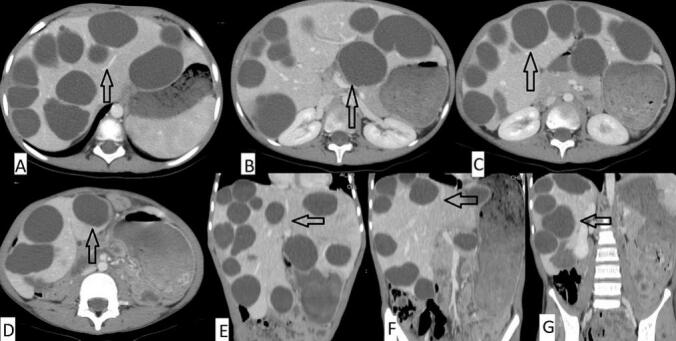
Presents axial CT images (A-D) and coronal (*E*-G) revealed an enlarged liver measuring 16 cm with multiple hypodense non-enhancing cystic lesions with thin enhancing walls in both lobes marked by black arrows, classified as 1CE. The largest hepatic cyst in segment II measured approximately 5.5 × 3.5 cm.

The imaging findings were consistent with hydatid disease, supported by the classic “water lily sign” in the lung lesions and multiple cystic lesions in the liver (Fig. 1, Fig. 2) According to the WHO-IWGE PNM classification, this case is categorized as P2N2M0, reflecting multiple liver (P2) and bilateral lung (N2) cysts without involvement of other organs (M0). This classification supports the decision for staged, organ-specific management. Brain CT scan was normal. The patient underwent a minimally invasive hybrid procedure involving a mini right thoracotomy for precise cyst excision. Eight hydatid cysts were removed from the right upper and lower lung lobes. The surgery included capitonage and hypertonic saline irrigation, using LigaSure for adhesion release and cyst deroofing. Postoperatively, two chest tubes were placed, and the patient showed significant clinical improvement.

**Fig. 3:**
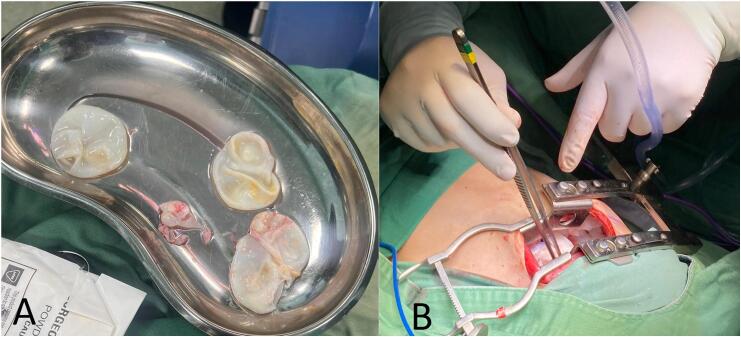
(A) Surgical tray displaying multiple hydatid cysts removed from the patient during the procedure. The cysts show the typical features of *Echinococcus* hydatid cysts, including translucent germinal layers and visible daughter cysts. (B) Intraoperative image showing the excision of hydatid cysts through a posterolateral mini thoracotomy incision.

Two weeks later, the patient underwent a minimally invasive left thoracotomy combined with VATS. Five hydatid cysts were excised from the left lung using similar techniques, including hypertonic saline injection and capitonage (Fig. 3). The VATS procedure utilized a two-port technique with incisions placed in the 5th and 7th intercostal spaces. A 10 mm camera port and a 5 mm working port were used. The lung was deflated to facilitate cyst exposure, and careful dissection was performed using an endoscopic grasper and energy device to avoid spillage. The cyst cavity was irrigated with hypertonic saline. The surgery proceeded without complications, and the patient continued to improve with regular physiotherapy and ambulation.

**Fig. 4:**
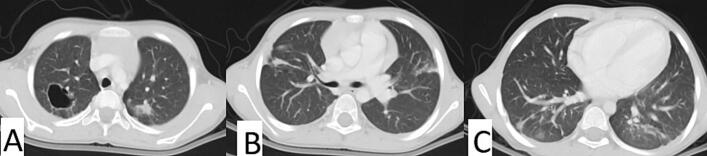
Shows axial CT images (A-C) taken at different levels of the lungs, four months post-surgery. The images reveal no recurrence of cystic lesions, with clear lung fields and normal pulmonary structures visible.

He was discharged in good general condition with stable vital signs and no respiratory distress. Discharge medications included albendazole for three months, with biweekly liver function tests to monitor for potential hepatotoxicity. Follow-up plans involved regular outpatient visits to monitor for any signs of recurrence or complications. A repeated CT scan after four months showed complete resolution of the lung cysts (Fig. 4). Then he was scheduled for further evaluation and potential liver cyst excision.

**Fig. 5:**
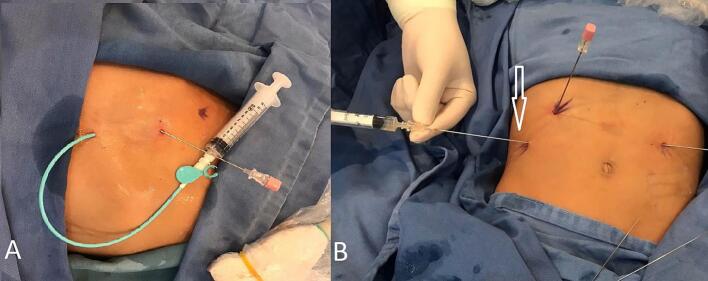
A, B Intraoperative image illustrating the PAIR (Puncture, Aspiration, Instillation, and *Re*-aspiration) procedure being performed to reduce hepatic cyst burden.

Given the patient's extensive burden of hepatic cysts, we began by performing multiple PAIR (Puncture, Aspiration, Instillation, and Reaspiration) procedures to significantly reduce both the number and size of the cysts. This minimally invasive approach, as shown in Fig. 5, helped decrease the risk of complications such as rupture or infection. Follow-up evaluations were favorable, indicating that the initial interventions not only lowered the overall cyst load but also improved the patient's readiness for subsequent surgical management. As a result, the patient is now scheduled for surgical removal of the remaining hepatic cysts. By taking this staged approach, we aim to ensure the safest possible surgical environment and achieve more successful long-term outcomes.

## 3 Discussion

Echinococcosis, primarily caused by *Echinococcus granulosus*, leads to cystic echinococcosis (CE). In our 8-year-old patient with multiple liver and lung cysts, dogs act as definitive hosts and livestock as intermediate hosts, while humans become infected by ingesting eggs from dog feces. These eggs release oncospheres that migrate to organs, forming hydatid cysts filled with infectious protoscolices. Although these cysts grow slowly, they pose serious health risks if untreated.

Diagnosis relies on epidemiological factors such as coming from an endemic region along with characteristic imaging features and immunodiagnostic methods [6]. In this case, symptoms like chronic cough, weight loss, and liver enlargement initially suggested tuberculosis, but lack of exposure and travel history made this less likely. Elevated eosinophil counts, increased C-reactive protein levels, and positive *Echinococcus* antibodies pointed toward hydatid disease, confirmed by imaging showing multiple cystic lesions in the lungs and liver.

Management typically involves surgery and antiparasitic therapy, with postoperative treatment significantly reducing recurrence rates [7]. Surgical intervention depends on cyst size, location, and complications. For large pulmonary cysts, thoracotomy is preferred over thoracoscopic methods due to lower risks of complications like intrabronchial rupture and bronchopleural fistulas [8,9]. In this patient, a minimally invasive mini thoracotomy was performed to remove lung cysts.

The decision to address pulmonary hydatidosis before hepatic cysts was based on the risk of cyst rupture into the bronchial tree, which can lead to life-threatening complications such as anaphylaxis or airway obstruction. Additionally, the child's respiratory symptoms were more clinically significant. PAIR was initially deferred due to the increased risk of dissemination in highly vascular hepatic cysts.

Hepatic cysts are managed through cystectomy or pericystectomy. Immediate surgery was not pursued for liver cysts due to the absence of rupture or biliary involvement. Complete cyst removal with a scolicidal agent is considered curative [7], also PAIR procedure is a minimally invasive technique for treating liver hydatid cysts, particularly those caused by *Echinococcus granulosus*. This method involves percutaneous puncture of the cyst under ultrasound or CT guidance, aspiration of the cystic fluid, injection of a scolicidal agent to inactivate the parasites, and re-aspiration to remove the solution. Studies have demonstrated that PAIR, when combined with albendazole or mebendazole therapy, is effective in managing hepatic cystic echinococcosis, offering a less invasive alternative to traditional surgical methods [18].

Albendazole is crucial as adjunctive therapy due to its superior penetration of the cyst membrane [7]. It was prescribed postoperatively to prevent recurrence, essential for managing residual cysts and preventing new formation.

Postoperative morbidity is higher in patients with both pulmonary and hepatic cysts, especially when complicated by pleural involvement. Complicated cases may have morbidity rates up to 16.3 % [10]. However, our patient had a smooth recovery with no significant complications, thanks to prompt and well-planned surgical intervention.

Mortality in hydatid disease is generally low, ranging from 0 % to 2 %, slightly higher in complicated cases [10]. Recurrence rates are also low, between 1.5 % and 2 %, particularly with appropriate postoperative therapy. In this case, albendazole was administered for two months, effectively preventing recurrence [10].

Although rare, complications of hepatic hydatid cysts can be life-threatening, including rupture which occurs in 20–50 % of cases and infection, seen in 5–8 % [17]. Ruptures are classified into communicating (most common at 15 %), contained (12 %), and direct (6 %). Another complication is the mass effect on adjacent structures [6].

Through a comprehensive review of the English-language literature, we identified six published studies [[11], [12], [13], [14], [15], [16]] related to hepatopulmonary hydatidosis in the pediatric age group. These studies included a total of six patients, all of whom shared several clinical features that led to the diagnosis. The distinguishing feature of our case is that the CT scan revealed multiple CE3a cystic lesions in both lungs, whereas in the previously mentioned studies, the cysts were confined to one lung. As a result, the patient underwent two consecutive surgeries for lungs cysts excision with no complications.

In our literature review, we concluded that despite hepatopulmonary hydatidosis being less common in children, it poses significant diagnostic and therapeutic challenges, particularly in cases involving multiple organs. The reviewed cases highlight the importance of thorough diagnostic evaluations, a combination of medical and surgical treatments.

Given the zoonotic and potentially familial transmission of hydatid disease, especially in endemic rural areas, the patient's household contacts were advised to undergo screening via abdominal ultrasound and serological testing. Education was provided on proper hygiene, deworming of pets, and avoiding consumption of contaminated food or water.

In the context of hydatid disease, presenting with an exceptionally high number of cysts is exceedingly rare. Our patient had 35 hydatid cysts distributed between the lungs and liver, which represents one of the highest numbers reported in the pediatric population. Most cases in the literature involve a limited number of cysts localized to a single organ. Such extensive multiorgan involvement not only poses significant challenges in management but also underscores the rarity of this case. This highlights the importance of individualized treatment plans and a multidisciplinary approach in handling severe presentations of hydatid disease.

## 4 Conclusion

This case underscores the rarity and complexity of extensive hepatopulmonary hydatidosis in an 8-year-old patient, presenting with over 12 cysts in the lungs and more than 20 in the liver; a highly unusual finding for a patient of this age. Our approach employed a minimally invasive hybrid technique, utilizing small incisions for cyst excision, which proved essential in effectively managing such a high cyst count while minimizing postoperative complications. This technique highlights the benefits of minimally invasive procedures in complex pediatric hydatid cases, facilitating recovery and reducing morbidity. The case exemplifies the importance of careful surgical planning and a multidisciplinary approach in treating high-burden multi-organ hydatidosis, providing valuable insights into the management of rare pediatric echinococcosis.

## Patient consent statement

Written informed consent was obtained from the patient's parents/legal guardian for publication and any accompanying images. A copy of the written consent is available for review by the Editor-in-Chief of this journal on request.

## Ethical approval

This case report was reviewed by the Institutional Review Board (IRB) and was deemed exempt from formal ethical approval. The exemption was granted because the report is a retrospective analysis of a single patient's clinical course, does not involve new research on human subjects, and all patient data has been anonymized.

## Funding

The authors have not received any payments or services, either directly or indirectly from a third party in support of any aspect of this work.

## Author contribution

Salahaldeen Deeb, Dana Sayyedahmad, Zaid Sharif and Sireen Manasrah contributed to writing the first draft and editing of the first draft and writing the final manuscript.

Yousef Abuasbeh and Hussein Abusharkh performed the surgery of the patient and provided the necessary data.

Yousef Abuasbeh supervised the project.

All authors contributed to the article and approved the submitted version.

## Guarantor

Salahaldeen Deeb.

## Research registration number

Not applicable.

## Conflict of interest statement

The authors have no conflicts of interest to disclose.

## Data Availability

Data sharing is not applicable to this article as no new data were created or analyzed in this study.
